# Gas Chromatography/Atmospheric Pressure Chemical Ionization-Fourier Transform Ion Cyclotron Resonance Mass Spectrometry of Pyrolysis Oil from German Brown Coal

**DOI:** 10.1155/2016/5960916

**Published:** 2016-03-15

**Authors:** Jan Zuber, Marius M. Kroll, Philipp Rathsack, Matthias Otto

**Affiliations:** ^1^Institute of Analytical Chemistry, TU Bergakademie Freiberg, 09599 Freiberg, Germany; ^2^German Centre for Energy Resources, Reiche Zeche, Fuchsmuehlenweg 9, 09599 Freiberg, Germany

## Abstract

Pyrolysis oil from the slow pyrolysis of German brown coal from Schöningen, obtained at a temperature of 500°C, was separated and analyzed using hyphenation of gas chromatography with an atmospheric pressure chemical ionization source operated in negative ion mode and Fourier transform ion cyclotron resonance mass spectrometry (GC-APCI-FT-ICR-MS). Development of this ultrahigh-resolving analysis method is described, that is, optimization of specific GC and APCI parameters and performed data processing. The advantages of GC-APCI-FT-ICR-MS hyphenation, for example, soft ionization, ultrahigh-resolving detection, and most important isomer separation, were demonstrated for the sample liquid. For instance, it was possible to separate and identify nine different propylphenol, ethylmethylphenol, and trimethylphenol isomers. Furthermore, homologous series of different acids, for example, alkyl and alkylene carboxylic acids, were verified, as well as homologous series of alkyl phenols, alkyl dihydroxy benzenes, and alkoxy alkyl phenols.

## 1. Introduction

The upcoming depletion of fossil carbon resources, like crude oil and natural gas, forces all oil- and gas-consuming industries to scout for suitable substitutes. Pyrolysis oil is such resources, produced from organic material [1, 2]. Generally speaking, in the process of pyrolysis, molecules of higher molecular weight are thermally decomposed into smaller molecules in an oxygen-free atmosphere [3]. During this process, a great variety of chemical reactions, such as elimination, cracking, isomerization, and rearrangements reactions, take place [4], leading to ultracomplex product mixtures. Potential feedstocks are carbon-rich materials, for instance coal [5–13], biomass [14–17] or scrap tyres [18, 19]. Coal pyrolysates are mainly composed of aliphatic and aromatic compounds, with varying heteroatomic content (i.e., S, O, and N) [9–12]. The hyphenation of gas chromatography (GC) with high-resolution mass spectrometry (MS) represents a method to analyze these complex mixtures in depth [20]. In general, the volatile parts of oil are separated by means of gas chromatography and subsequently identified by mass spectrometry. This hyphenation can be applied, for example, to analyze pyrolysis products with different polarities [21–23]. With this knowledge, much more efficient oil reprocessing and choice of the pyrolysis oil application site are possible.

The contents of the pyrolysis oil can be ionized extensively by using a sensitive and selective ion source, like an atmospheric pressure chemical ionization (APCI) source or an atmospheric pressure laser ionization (APLI) source, coupled to an ultrahigh-resolving (UHR) mass analyzer, such as the Fourier transform ion cyclotron resonance mass spectrometer (FT-ICR-MS). APCI is a special form of chemical ionization (CI), where a corona needle current regulated high voltage direct current (HV-DC) gradient is employed to generate a corona plasma. This plasma causes the formation of reactive species, which ionize the analyte molecules in a subsequent secondary ionization process [24]. In comparison, APLI is an ionization technique which uses pulsed laser light to produce molecular ions [25]. Both ionization methods are much more sensitive and selective than the electron ionization (EI) that is commonly used for GC-MS hyphenation [24–33]. APCI is mainly adopted to analyze compounds with higher polarity, such as phosphoric acid esters and carbamates [24]. In contrast, APLI is a technique to analyze preferably nonpolar compounds, like polycyclic aromatic hydrocarbons (PAHs) [25, 26]. According to the literature, a compound quantification with these soft ionization methods up to 5 fg is possible [26].

In a previous paper, our research group was able to demonstrate the potential of the FT-ICR-MS on pyrolysis oil characterization, using an electrospray ionization (ESI) source [34]. We could identify different single compounds and compound classes for a representative pyrolysis oil from a German brown coal. Main disadvantage of this analysis technique was its missing chromatographic separation. Hence, a fractionation of compounds with the same exact molecular formula was not possible. This drawback was also acknowledged by other research groups [32, 35]. Theoretically, the hyphenation of GC with FT-ICR-MS using a soft APCI ion source should provide an opportunity to separate and detect molecules with the same exact monoisotopic mass with an ultrahigh mass resolution.

The aim of this study is the development of a method for the analysis of a pyrolysis liquid from German brown coal by hyphenation of GC with FT-ICR-MS using an APCI source. A representative mixture of standard compounds (RMSC) was used for determination of optimum parameters for APCI ion source and GC. Results from APCI were analyzed based on type, frequency, and intensity of assigned molecular formulas. The potential of the developed method, that is, soft ionization, isomeric separation, and ultrahigh mass detection, was demonstrated for the actual pyrolysis liquid sample. To the best of our knowledge, the applied GC-APCI-FT-ICR-MS analyses represent the first application of this analytical method for characterization of a pyrolysis liquid from a German brown coal.

## 2. Materials and Methods

### 2.1. Preparation of Pyrolysis Oil Sample

A pyrolysis liquid from German brown coal pyrolysis was used for applicability tests and will be denoted as S500 during the discussion. The sample was produced by means of a fixed bed reactor in batch mode. Pulverized coal was filled into the tubular reactor and heated by an external oven to the designated temperature of 500°C. Pyrolysis products were trapped in two subsequent cooled traps (−18°C). The first trap was a packed bed and the second a solvent (tetrahydrofuran (THF)) filled trap. After each experiment, the first trap was flushed with solvent to release the oil. The extract was combined with the solvent from the second trap and the solvent was separated from the oil by distillation.

The sample was weighted in an Erlenmeyer flask and diluted with benzene to yield a mass concentration of 100 g·L^−1^. Hereinafter, the sample was shaken at 20°C and 800 min^−1^ for 30 min in a Vortex Shaker and centrifuged for 10 min at 10,000 G. Supernatant solution was separated from the undissolved pellet and transferred to amber vials.

Reference compounds for the RMSC were weighted into amber glass vials and solved in methyl acetate to yield a mass concentration of 1 g·L^−1^ per compound. All reference compounds were purchased from Sigma-Aldrich either as analytical standard or as highest purity available. The RMSC was composed of aromatic (e.g., benzene) and heterocyclic compounds (e.g., thiophene), with a varying number of alkyl groups. Its detailed composition is shown in the Supplementary Material (see Section S1 in Supplementary Material available online at http://dx.doi.org/10.1155/2016/5960916).

Benzene and methyl acetate were also purchased from Sigma-Aldrich and both of 99.8% purity.

### 2.2. Instrumental Parameters

The chromatographic separation of pyrolysis oil constituents was performed using Bruker Daltonics 450-GC with a VARIAN CP-8400 autosampler. The gas chromatograph was equipped with a SUPELCOWAX 10 capillary column (*L* = 30 m, *d*_*c*_ = 0.25 mm, and *d*_*f*_ = 0.25 *μ*m) from Supelco, which has a polyethylene glycol stationary phase. The GC temperature program started at a temperature of 100°C, which was held for 2 min, then ramped to 250°C, at a constant heating rate of 5 K·min^−1^, and finally held at this temperature for 30 min, resulting in an overall analysis time of 62 min. GC parameters gas flow, injection volume, and split-radio were optimized during the method development process, as presented hereinafter.

A 15 T solariX FT-ICR-MS from Bruker Daltonics was hyphenated, equipped with a Bruker Daltonics GC-APLI/D Source, and operated in negative ion mode, with a scan range from 46.06 to 500 Da, a transient length of 2 M words, and 300 ms ion accumulation time. These settings resulted in a scan time of 1.003 s per single spectrum and hence a data acquisition rate of 0.997 Hz. Furthermore, the corona needle current and capillary voltage were also optimized during the method development process. Not explicitly optimized parameters of the APCI ionization source were chosen as follows: end-plate-offset: 0 V, dry gas flow: 1.5 L·min^−1^, dry gas temperature: 250°C, nebulizer gas pressure: 3.0 bar, and vaporizer temperature: 350°C.

External calibration of the GC-APCI-FT-ICR-MS analyses was performed using a liquid standard that was composed of* n*-hexane, 250 pmol trifluoroacetic acid (TFA), 50 pmol heptafluorobutyric acid (HFBA), and 16 mmol dichloromethane (DCM). The standard solution was introduced using a Thermo Scientific Dionex UltiMate 3000 HPLC pump at a constant flow rate of 80 *μ*L·min^−1^.

### 2.3. Experimental Design

Creation and evaluation of the experimental design were performed using* Statgraphics Centurion XVI*. However, an experimental design was only necessary for the optimization of the APCI parameters that were varied simultaneously. Therefore, a 3-level full factorial design was applied. Every single optimization experiment was analyzed according to the intensity values of specific compounds of the RMSC that were found in the extracted ion chromatograms (EICs). For evaluating the optimization, the following mass traces were examined: (*m/z*) = 93.034588, (*m/z*) = 107.050238, (*m/z*) = 109.029503, (*m/z*) = 121.065888, (*m/z*) = 123.045153, (*m/z*) = 151.040068, (*m/z*) = 151.076453, (*m/z*) = 163.076453, and (*m/z*) = 165.092103. We assumed all investigated compounds to be present as [M − H]^−^ molecular ions. This assumption is primarily based on the high mass accuracy of the FT-ICR-MS, which supports the general existence of the [M − H]^−^ molecular ions with a high degree of certainty. In case of the APCI optimization, the intensity sum of all investigated peaks in the specific EICs was used as validation factor. In comparison to the APCI parameter optimization, GC parameters were optimized successively. The validation of GC optimization experiments was conducted by comparing peak intensities, peak areas, and observed peak separation of the single compounds in the above described mass traces.

### 2.4. Data Processing

The data sets were calibrated and processed with an automated in-house tool, using Bruker Daltonics software* Data Analysis* (*4.1 SP1*). The tool was created with* Microsoft Visual Basic*. Its main task was the calibration and thus (*m/z*) correction of every acquired mass spectrum in each time segment. As described before, calibration was performed using a liquid standard, composed of* n*-hexane, TFA, HFBA, and DCM. These standard compounds themselves formed characteristic molecular ions during the ionization process that were present throughout the analysis and used as reference. This resulted in a processing output, where for each time segment peak and molecular formula lists were created. More precisely, the peak lists contained information about the detected analyte signals in the specific time section with their (*m/z*)-ratios, intensities, resolution, and signal-to-noise-ratios (*S/N*). Compared to that, molecular formula lists comprised molecular formula suggestions based on the detected (*m/z*)-ratios. Furthermore, mass errors and standard deviations to the assigned molecular formulas were listed. Application of this data processing method reduced the amount of data from approximately 77.2 GB (unprocessed raw data) to 55.3 MB (entirety of output files).

**Figure 1:**
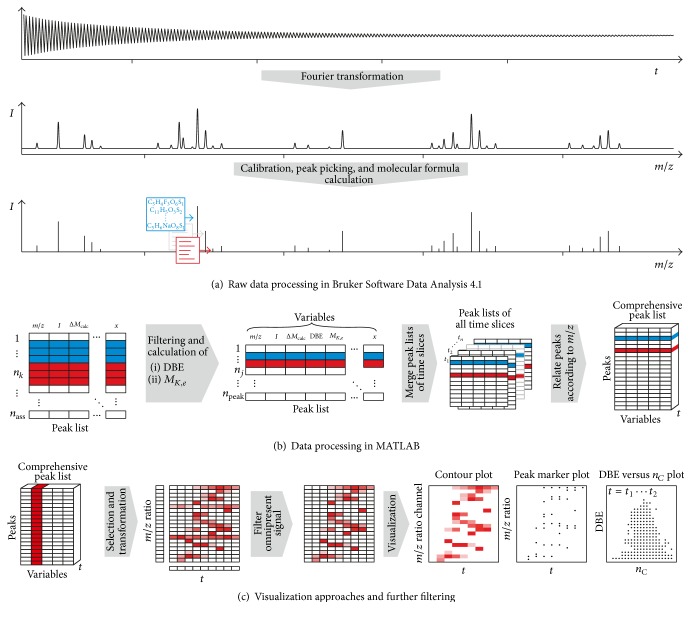
Flow diagram of performed data processing.

In the next processing step, the acquired molecular formula lists were processed with in-house* MATLAB* scripts; that is, the peak and molecular formula lists were loaded into* MATLAB R2015b* as  .csv-files and different figures for the experiment evaluation were created. By filtering out all calibrant signals that were present throughout the analysis, the plots shown in Section 3 were generated. A flow sheet of the conducted data processing is visualized in Figure 1.

For further information on the used molecular ions for data calibration, see Section S2 in the Supplementary Material. Screen shots of the calibration and processing tool and its outcome files are presented in the Supplementary Material (see Section S3) as well.

## 3. Results and Discussion

### 3.1. Optimization of GC and APCI Parameters

The optimization of GC and APCI source parameters was conducted using a mix of standard compounds, representative for aromatic pyrolysis oil constituents (RMSC). Main goal of the GC optimization was to improve three specific parameters: Gas flow, injection volume, and split-ratio. Their values were varied and optimized in case of the gas flow from 0.6 to 1.2 mL·min^−1^ in 0.2 mL·min^−1^ steps, in case of the injection volume from 1.0 to 2.5 *μ*L in 0.5 *μ*L steps, and in case of the split-ratio from splitless injection to 1 : 20 in five single experiments (splitless, 1 : 2, 1 : 5, 1 : 10, and 1 : 20). The EICs of the RMSC were evaluated in terms of signal intensity to determine the ideal GC parameters. Furthermore, the baseline separation of EIC peaks of compounds with the same (*m/z*)-ratio (e.g.,* o*- and* m*-cresol ((*m/z*) = 107.050238)) and the areas under the EIC peaks were analyzed by integration, using a Gaussian filter for peak shape fitting. The mass traces used for experiment evaluation of the GC optimization were chosen as described before in the experimental section.

According to the applied analysis, the following GC parameters were determined as ideal: gas flow: 0.8 mL·min^−1^, injection volume: 1.0 *μ*L, and splitless injection. Considering the intensity values, baseline peak separation, and peak areas of the single components in the EICs, these parameters yielded the highest intensities and peak areas and thus were applied to the actual sample investigation.

The optimization of the APCI parameters was performed similar to the GC optimization, using the same mix of standard compounds (RMSC). The main goal of this optimization was to improve two parameters of the APCI source simultaneously: corona needle current and capillary voltage. The corona needle current is essential to generate the plasma, which itself is required to induce the ionization process. Capillary voltage is an electrical potential that is applied to transport the produced ions to the skimmer and ion funnel [20]. Their values were varied in case of the corona needle current from 10 to 15 *μ*A in 2.5 *μ*A steps and in case of the capillary voltage from +100 V to +1100 V in 500 V steps. Analogous to a 3-level full factorial design, nine randomized experiments were conducted.

According to the optimization, the following ideal values for corona needle current and capillary voltage were determined: corona needle current 15 *μ*A and capillary voltage +100 V. The highest overall intensity sum values as well as the highest single intensity values for most of the EIC peaks of the individual components were obtained at these parameters.

Altogether, the optimization of GC and APCI parameters helped to shape an UHR analysis method that was applied hereinafter to an actual pyrolysis liquid sample, namely, S500.

### 3.2. Analysis of Representative Pyrolysis Liquid Sample

The reproducibility of conducted experiments was investigated by analyzing pyrolysis oil S500 three times successively. In seven different EICs, only baseline separated peaks were selected and peak shape fitted using a Gaussian filter. More precisely, following mass traces were evaluated: (*m/z*) = 93.034588, (*m/z*) = 107.050238, (*m/z*) = 109.029503, (*m/z*) = 121.065888, (*m/z*) = 123.045153, (*m/z*) = 135.081539, and (*m/z*) = 151.040068. Peak shape fitted peaks were analyzed based on peak intensity, peak area, and* S/N*. For each assessment parameter, a mean value of the three sample measurements was computed, separately for each peak and overall for all investigated peaks as well. Hereinafter, the absolute and relative standard deviation (RSD) of every measurement, according to the deviation from calculated mean values, was determined. Thereby, an averaged RSD for all examined peaks of 15.68% in terms of peak intensity (range of 0.30–45.33%), 24.47% in terms of peak area (range of 0.69–95.20%), and 19.49% in terms of* S/N* (range of 2.18–62.97%) was obtained. Hence, the developed analysis method generates qualitatively reproducible results. In particular, RSD values of 0.30% at (*m/z*) = 135.081539 in terms of signal intensity or 0.69% at (*m/z*) = 151.040068 in terms of peak area demonstrate the good qualitative reproducibility. Nonetheless, reproducible quantitative statements are not feasible at this point, due to the high variance in received RSD values, particularly in terms of peak area and* S/N*. Better RSD values and minimized RSD variances could be achieved if experiments with added internal standards are conducted.

**Figure 2:**
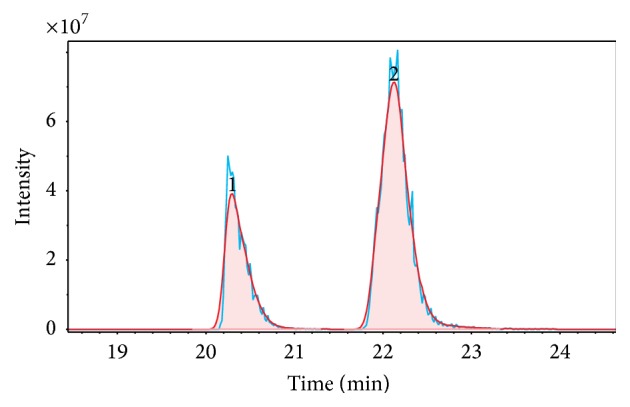
EIC at (*m/z*) = 107.050238. Two peaks can be assigned to two different cresol isomers. Sample signals are presented in blue. Gaussian peak shape fitted peaks and integrated peak areas are presented in red.

**Figure 3:**
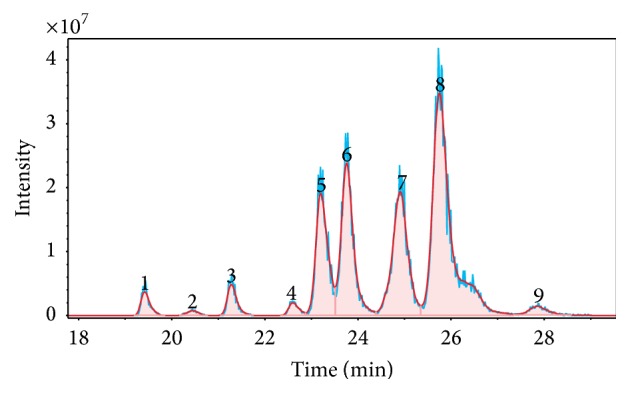
EIC at (*m/z*) = 135.081539. Nine peaks can be assigned to nine different propylphenol (e.g., cumenol), ethylmethylphenol, and trimethylphenol (e.g., mesitol) isomers.

Since FT-ICR-MS is a high-resolving analysis technique, it allows very accurate mass measurements and molecular formulas can be derived from the exact masses. The number of potential compounds for one mass trace depends on mass accuracy and the elements (and isotopes) taken into account. In conjunction with GC, it was possible to demonstrate a significant advantage of the GC-MS hyphenation in comparison to isolated MS experiments, that is, isomeric separation. Two EIC examples, exhibiting a successful isomeric separation, are illustrated in Figures 2 and 3. Plots shown were directly exported from the MS integrated software. In these chromatograms, actual sample signals are presented in blue and corresponding Gaussian peak shape fitted signals in red.

The first example, illustrated in Figure 2, presents the EIC at (*m/z*) = 107.050238. According to our mix of standard compounds, RMSC, this (*m/z*)-ratio refers to two different methylphenols (cresols),* o*- and* m*-cresol. Both peaks were baseline separated.

A second example for the successful isomeric separation of liquid oil compounds was observed at (*m/z*) = 135.081539 (see Figure 3). This (*m/z*)-ratio can be assigned to propylphenols (*n*- and* iso*-(cumenol)), ethylmethylphenol, and/or trimethylphenols, such as mesitol (2,4,6-trimethylphenol). Altogether, we observed nine different peaks and hence nine compounds in this mass trace.

Considering the presented EICs, the developed GC-APCI-FT-ICR-MS method finally gives a possibility to separate compounds with the same exact molecular mass and thus the same exact (*m/z*)-ratio and detect them with an ultrahigh-resolving mass spectrometric analyzer. Therefore, the established GC-APCI-FT-ICR-MS hyphenation can be considered an UHR analysis method.

**Figure 4:**
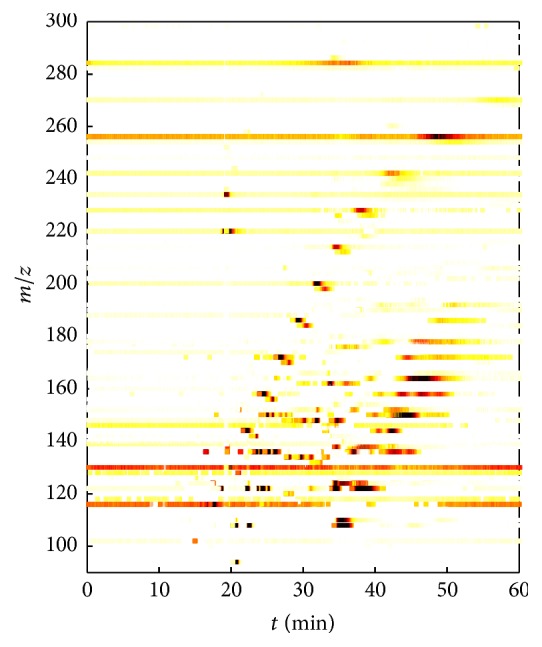
Spectrochromatogram of sample S500. The (*m/z*) values are plotted against the retention time and observed relative intensities are visualized color-coded ones (white, low intensity; red, medium intensity; black, high intensity).

Figure 4 shows the spectrochromatogram of the GC-APCI-MS analysis of sample S500, during the overall analysis time (in min) and exhibits (*m/z*)-ratios in the range of 90–300 Da. Observed relative intensity values are illustrated as color-coded ones (white: low intensity, red: medium intensity, and black: high intensity). According to Figure 4, we could verify different compounds of the RMSC in this plot, for example, phenol ((*m/z*) = 93.034588),* o*- and* m*-cresol ((*m/z*) = 107.050238), ethyl- and dimethylphenols ((*m/z*) = 121.065888), vanillin ((*m/z*) = 151.040068), and different eugenols (*cis*- and* trans*-isoeugenol, eugenol) ((*m/z*) = 163.076453). Molecular ions, which were detected throughout the analysis, can be mainly attributed to additionally added reference compounds (*n*-hexane, TFA, HFBA, and DCM). Also ubiquitous signals, whose* m/z* values correspond to analytes of the pyrolysis liquid, were detected over the entire analysis time. For instance, a compound was observed at (*m/z*) = 255.232954, that was present in a* S/N* range slightly higher than the corresponding threshold value (*S/N* = 5). Nonetheless, although an omnipresent background was noticed at these mass traces, clear peak maxima resulted when corresponding compounds eluted.

Furthermore, we observed deprotonated molecules, that varied in terms of (*m/z*)-ratios by 14.015650 Da. This refers to an increasing number of CH_2_ groups and hence longer alkyl chains, resulting in a homologous series of different compounds. We were able to identify six different homologous series of compound classes, according to the observed (*m/z*)-ratios: saturated monocarboxylic acids (alkyl carboxylic acids), unsaturated monocarboxylic acids (alkylene carboxylic acids), dicarboxylic acids, alkyl phenols, alkyl dihydroxy benzenes, and alkoxy alkyl phenols. Corresponding alkyl dihydroxy benzenes and alkoxy alkyl phenols have same exact molecular masses and thus same exact (*m/z*)-ratios, resulting in a detection in the same EICs.

**Figure 5:**
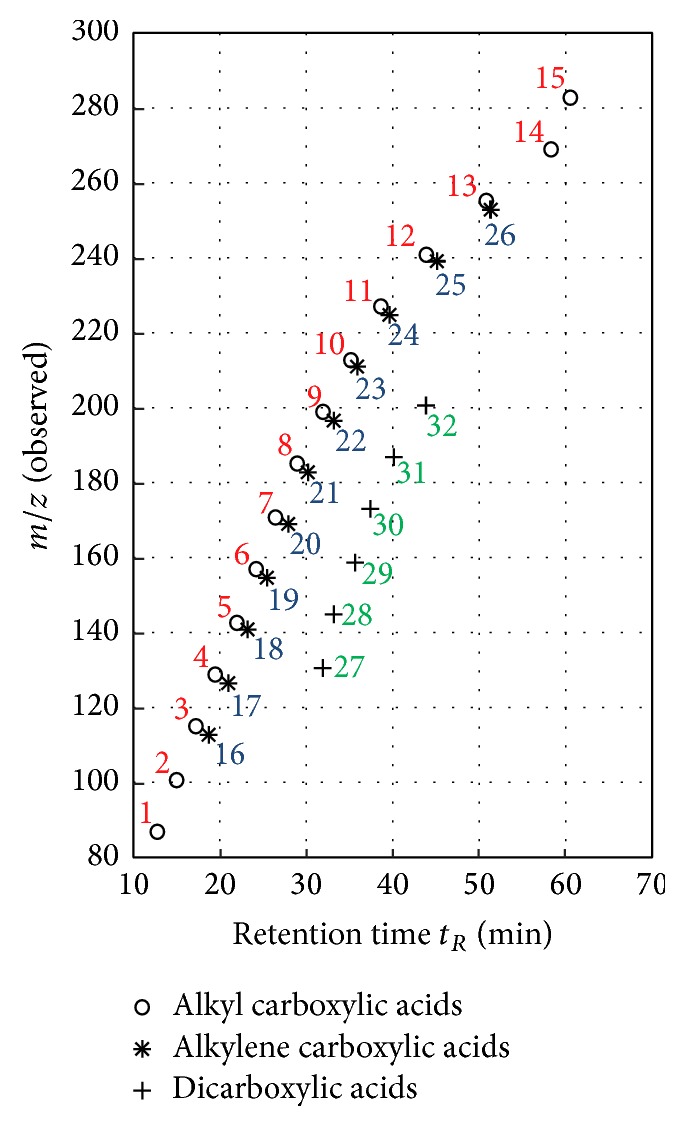
Scatter plot of identified alkyl carboxylic acids (red), alkylene carboxylic acids (blue), and dicarboxylic acids (green). 1: butanoic acid, 15: octadecanoic acid; 16: hexenoic acid, 26: hexadecenoic acid; 27: pentanedioic acid, 32: decanedioic acid (see also Table 1).

Figure 5 illustrates the (*m/z*)-ratios and retention times (in min) of all allocated saturated and unsaturated monocarboxylic acids and dicarboxylic acids. As shown in this figure, we could discern 15 alkyl carboxylic acids with a chain length from C_4_ to C_18_, 11 alkylene carboxylic acids with a chain length from C_6_ to C_16_, and six dicarboxylic acids with a chain length from C_5_ to C_10_.  Table 1 presents an overview of all assigned saturated and unsaturated monocarboxylic acids and dicarboxylic acids, exhibiting assigned compound classes, assigned compounds, assigned number (see Figure 5), retention time *t*_*R*_ in min (represented as confidence interval), theoretical calculated (*m/z*)-ratios, observed (*m/z*)-ratios, (*m/z*) errors in mDa, and (*m/z*) errors in ppm. Calculated (*m/z*) errors varied in case of the alkyl carboxylic acids from 0.096 to 0.427 ppm (mean value: 0.210 ppm), the alkylene carboxylic acids from 0.021 to 0.367 ppm (mean value: 0.165 ppm), and the dicarboxylic acids from 0.038 to 0.107 ppm (mean value: 0.070 ppm). Hence, the applied assignment of observed (*m/z*)-ratios to the described compounds is most accurate for the five dicarboxylic acids but also quite accurate for the other two compound classes.

**Figure 6:**
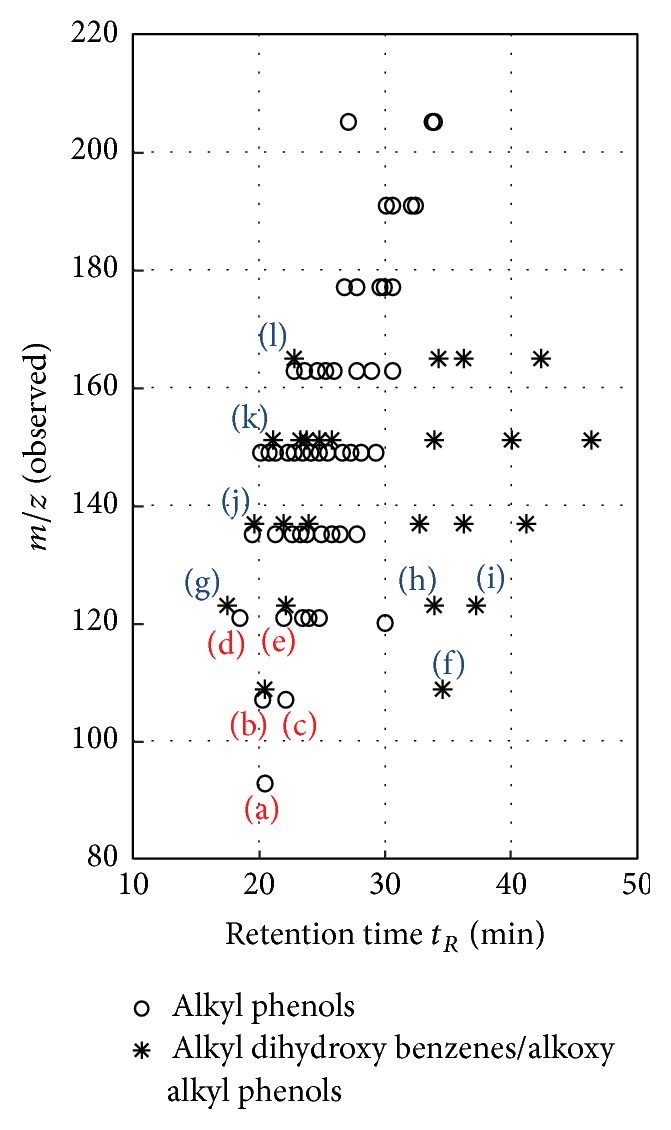
Scatter plot of assigned alkyl phenols, alkyl dihydroxy benzenes, and alkoxyl alkyl phenols. Identified components of the RMSC are colored red for alkyl phenols and blue for alkyl dihydroxy benzenes/alkoxyl alkyl phenols. (a) Phenol, (b)* o*-cresol, (c)* m*-cresol, (d) 2,6-dimethylphenol, (e) 2-ethylphenol, (f) hydroquinone, (g) guaiacol, (h) 4-methylcatechol, (i) 2-methylhydroquinone, (j) 4-methylguaiacol, (k) 4-ethylguaiacol, and (l) 4-propylguaiacol.

Figure 6 illustrates the observed (*m/z*)-ratios and retention times (in min) for the identified formulas of alkyl phenols, alkyl dihydroxy benzenes, and alkoxy alkyl phenols. We could presumably identify 50 different alkyl phenols in nine distinct mass traces and 24 different alkyl dihydroxy benzenes/alkoxy alkyl phenols in five distinct EICs. In accordance with the compounds of the RMSC, we were able to identify phenol (a) at (*m/z*) = 93.034588,* o*-cresol (b) and* m*-cresol (c) at (*m/z*) = 107.050238, and two dimethyl- and ethylphenols, 2,6-dimethylphenol (d), and 2-ethylphenol (e) at (*m/z*) = 121.065888. Higher (*m/z*) values, increasing by 14.015650 Da (mass of a CH_2_ group), indicating longer alkyl chains at the phenolic core, for example, trimethylphenol or tetramethylphenol, are plausible. Compounds of the RMSC were also verified for alkyl dihydroxy benzenes and alkoxy alkyl phenols as well. For instance, peaks at (*m/z*) = 109.029503 can be identified as hydroquinone (f), (*m/z*) = 123.045153 as guaiacol (g), 4-methylcatechol (h), and 2-methylhydroquinone (i), (*m/z*) = 137.060803 as 4-methylguaiacol (j), (*m/z*) = 151.076453 as 4-ethylguaiacol (k), and (*m/z*) = 165.092103 as 4-propylguaiacol (l). Higher (*m/z*) values for these proposed compound classes were not observed. An overview, such as Table 1 for the carboxylic acids, is not presented in the main part of this paper for alkyl phenols, alkyl dihydroxy benzenes, and alkoxy alkyl phenols, due to the high number of detected peaks and assignable compounds in the specific mass traces. Carboxylic acids were always present in the EICs with just one peak and hence could be evaluated more easily. However, the overview for alkyl phenols, alkyl dihydroxy benzenes, and alkoxy alkyl phenols can be found in the Supplementary Material (see Section S4).

Compared to pyrolysis liquid sample S500, retention times of alkyl dihydroxy benzenes, for example, 4-methylcatechol and 2-methylhydroquinone, and alkoxy alkyl phenols, for example, guaiacol and 4-methylguaiacol, were consistent with respect to the retention times of the RMSC. As shown in Figure 6, it was possible to separate and identify alkyl dihydroxy benzenes and alkoxy alkyl benzenes, regardless of their same exact molecular mass and (*m/z*)-ratio, by comparing their retention times. Additionally, compound clustering of alkyl dihydroxy benzenes and alkoxy alkyl phenols was observed, below and above a retention time of 32 min. Alkyl dihydroxy benzenes exhibit stronger interactions with the polar stationary phase than alkoxy alkyl phenols, because of the increased polarity of alkyl dihydroxy benzenes due to their additional second hydroxy group. Hence, alkoxy alkyl phenols elute earlier than the corresponding alkyl dihydroxy benzenes.

For a better assignment of detected compounds, apart from verified compounds of the RMSC, the concept of double bond equivalent (DBE) [13, 33, 34, 36] was applied. DBE is calculated using the molecular formula, while simplifying the manual search for potential compound structures. The DBE increases by one, if, for example, a double bond, a ring, a carbonyl group, or a carboxylic group is introduced into a structure.

**Figure 7:**
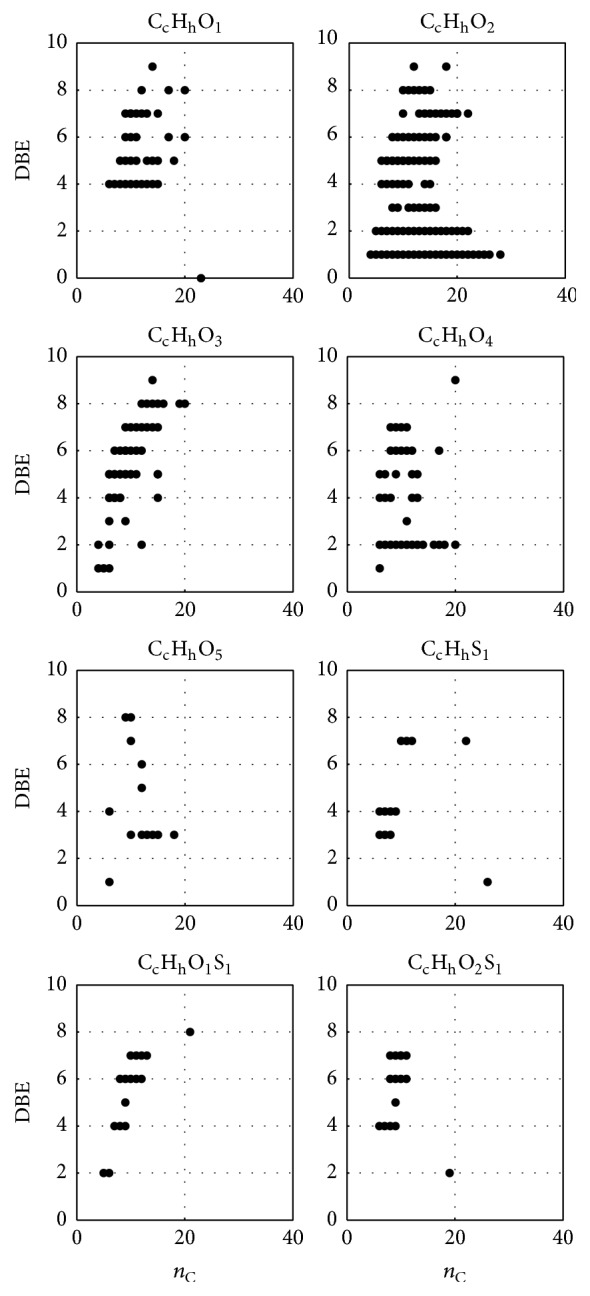
*n*_C_-DBE-plots of different compound classes of liquid sample S500.

Figure 7 shows the DBE plotted against the number of carbon atoms (*n*_C_) for eight different compound classes over the entire analysis time. Reference compounds that were ubiquitous, that is, calibrant signals, were filtered out to receive the illustrated figure. In general, predominantly oxygen- and/or sulphur-containing organic species were discovered in liquid sample S500, and hence only *n*_C_-DBE-plots of compound classes containing these elements are illustrated in Figure 7. Nitrogen-containing compounds were not observed, due to the applied negative ion mode and thus will not be evaluated further. The presented postulated structures are affiliated to reactions and structural changes of the raw coal during the pyrolysis process, according to existing knowledge about this class of substances.

Compounds of the RMSC were found in the *n*_C_-DBE-plot of compound class C_c_H_h_O_1_, such as phenol (a) (*n*_C_ = 6, DBE = 4),* o*- and* m*-cresol (b, c) (*n*_C_ = 7, DBE = 4), and 2,6-dimethylphenol (d) and 2-ethylphenol (e) (*n*_C_ = 8, DBE = 4) (see also Figure 6). Higher DBE values might refer to phenolic structures with annulated cycles, for example, cycloalkanes and cycloalkenes.

DBE values of 1 in compound class C_c_H_h_O_2_ match to assigned alkyl carboxylic acids and their corresponding esters as well. Besides previously determined monocarboxylic acids with *n*_C_  ≤ 18 (see Table 1), also compounds such as esters of cerotic acid (C_26_) or montanic acid (C_28_) were present. These compounds normally appear in montan wax; that is, they are constituents of the waxy contents of brown coal [37]. DBE values of 2 in compound class C_c_H_h_O_2_ were assigned to alkylene carboxylic acids and their esters, where compounds with *n*_C_ values between 5 and 22 were verifiable. Dicarboxylic acids and their corresponding esters were assigned to compound class C_c_H_h_O_4_ at a DBE of 2 with *n*_C_ values between 5 and 20.

Furthermore, alkyl dihydroxy benzenes and alkoxyl alkyl phenols were assigned to compound class C_c_H_h_O_2_ at a DBE of 4, for example, *n*_C_ of 6 matches to hydroquinone (f), *n*_C_ of 7 to guaiacol (g), 4-methylcatechol (h), and 2-methylhydroquinone (i), *n*_C_ of 8 to 4-methylguaiacol (j), *n*_C_ of 9 to 4-ethylguaiacol (k), and *n*_C_ of 10 to 4-propylguaiacol (l) (see also Figure 6).

Sulphur-containing compounds were assigned to the *n*_C_-DBE-plots of compound classes C_c_H_h_S_1_, C_c_H_h_O_1_S_1_, and C_c_H_h_O_2_S_1_. According to the plots of these compound classes, presumed compounds are alkyl derived thiophenes (C_c_H_h_S_1_, DBE = 3), mono carbonyl derived thiophenes (C_c_H_h_O_1_S_1_, DBE = 4), and benzothiophenes (C_c_H_h_O_1_S_1_, DBE = 7), as well as dihydroxy derived benzothiophenes (C_c_H_h_O_2_S_1_, DBE = 6) and mono carboxyl derived thiophenes (C_c_H_h_O_2_S_1_, DBE = 4) and benzothiophenes (C_c_H_h_O_2_S_1_, DBE = 7).

**Figure 8:**
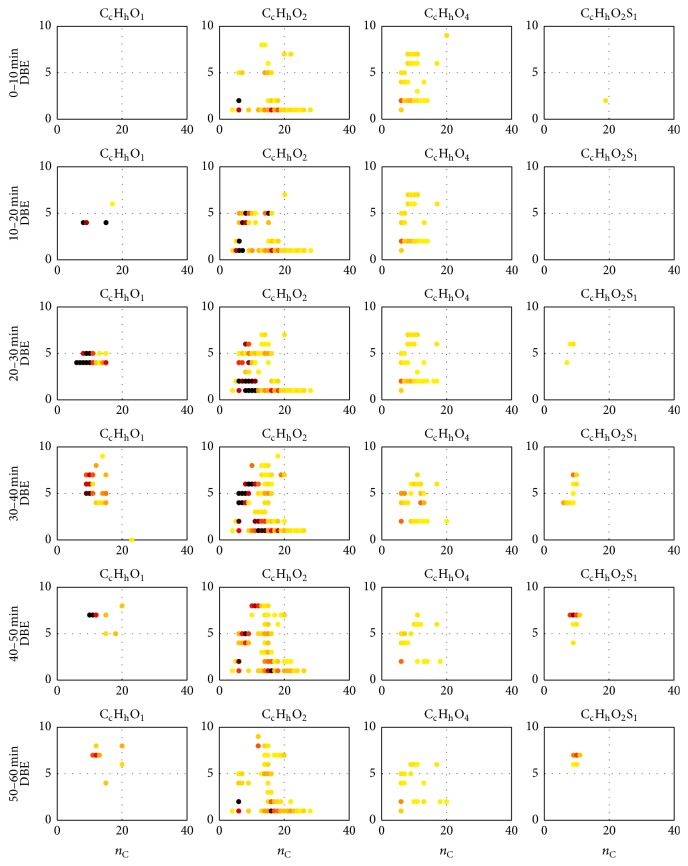
*n*_C_-DBE-plots for different time segments of compound classes C_c_H_h_O_1_, C_c_H_h_O_2_, C_c_H_h_O_4_, and C_c_H_h_O_2_S_1_. Observed relative intensities of detected compounds are visualized as color-coded ones (yellow: low intensity, red: medium intensity, and black: high intensity).

Retention time-depending statements are possible by using time separated *n*_C_-DBE-plots. The *n*_C_-DBE-*t*-plots of compound classes C_c_H_h_O_1_, C_c_H_h_O_2_, C_c_H_h_O_4_, and C_c_H_h_O_2_S_1_ are presented exemplarily in Figure 8. Detected compounds were pooled and plotted in 10 min analysis time segments. The time-dependent *n*_C_-DBE-plots of other evaluated compound classes are presented and interpreted in the Supplementary Material (see Section S5). The previously described filter procedure for the creation of *n*_C_-DBE-plots was also applied to generate the *n*_C_-DBE-*t*-plots.

In the illustrated *n*_C_-DBE-*t*-plots of compound class C_c_H_h_O_1_, no increase of *n*_C_ and DBE values with advancing analysis time was observed. As stated before, in this compound class phenol (a) and other alkyl phenols, like* o*- and* m*-cresol (b, c), can be found at *n*_C_ = 6, *n*_C_ = 7 and DBE = 4. These assigned compounds were mainly detected in a time range between 20 and 30 min. Compared to that, assigned saturated (DBE = 1) and unsaturated monocarboxylic acids (DBE = 2) and their corresponding esters in compound class C_c_H_h_O_2_ eluted throughout the analysis (0–60 min). Most likely, this could be attributed to the wider range of detected *n*_C_ values from 5 to 29 for saturated monocarboxylic acids and 5 to 22 for unsaturated monocarboxylic acids. Identified saturated dicarboxylic acids (DBE = 2) in class C_c_H_h_O_4_ were detected between 0 and 40 min. In general, a slight increase of observed *n*_C_ values with increasing analysis time was noticed in this class. Corresponding DBE values did not improve simultaneously.

Time-depending *n*_C_-DBE-plots of compound class C_c_H_h_O_2_S_1_ demonstrated an elution of assumed dihydroxy substituted benzothiophenic molecules (DBE = 6) between 20 and 60 min. In comparison, supposed thiophenes and benzothiophenes, containing a single carboxyl group (DBE = 4 and DBE = 7), were mainly detected in the second analysis half, that is, from 20–50 min and 30–60 min.

The *n*_C_-DBE-*t*-plots also illustrate the presence of ubiquitous signals at certain (*m/z*) values. For instance, in class C_c_H_h_O_2_, a signal leading to an assigned molecular formula with *n*_C_ = 6 and DBE = 5 was detected over the entire analysis time. Analogous to the spectrochromatogram (see Figure 4), varying intensity values were observed. The intensity of the sample compound is almost equal between 0 and 20 min, shows a maximum in the two subsequent time segments, and decreases thereafter until the end of the analysis. Hence, an approximate peak maximum identification is also possible from this type of illustration.

## 4. Conclusion

In this study, the hyphenation of gas chromatography (GC) with Fourier transform ion cyclotron resonance mass spectrometry (FT-ICR-MS), using an atmospheric pressure chemical ionization (APCI) source, was applied to pyrolysis oil, obtained at a pyrolysis temperature of 500°C from a German brown coal. The method development process included optimization of specific GC and APCI parameters, for example, by using an 3-level full factorial design. Furthermore, the processing of the obtained data was an essential part of the method development. Considering all applied method optimization steps, an ultrahigh-resolving (UHR) analysis method was developed. This method allows separating different isomers of a molecule and detecting them with ultrahigh (*m/z*) resolution. This isomer separation was exemplified, as shown in Figure 2, for the separation of two cresol isomers.

Furthermore, it was possible to demonstrate the high analytical potential of the applied GC-APCI-FT-ICR-MS hyphenation by verifying different homologous series in the liquid sample analysis. Observed compound classes, increasing by the number of CH_2_ groups, were alkyl carboxylic acids, alkylene carboxylic acids, dicarboxylic acids, alkyl phenols, alkyl dihydroxy benzenes, and alkoxy alkyl phenols.

For structural statements, a visualization of double bond equivalent (DBE) versus carbon number (*n*_C_) was applied, illustrated for the entire analysis time (see Figure 7) and for six different time segments (see Figure 8) as well. With the help of these plots, the GC retention time-depending appearance of specific compounds could be visualized. Mainly saturated and unsaturated monocarboxylic acids, as well as dicarboxylic acids, thiophenes, benzothiophenes, cycloalkanes, cycloalkenes, and PAH analogous structures, partially derived by oxygen- and sulphur-containing groups, were assumed and in parts identified. Due to the polar SUPELCOWAX 10 GC column, a clear separation of molecules of higher polarity was observed, such as saturated and unsaturated monocarboxylic acids, saturated dicarboxylic acids, and alkyl phenols. In particular, the observed separation of different carboxylic acid compounds and compound classes is economically interesting, considering their various potential applications [38].

According to the assigned compounds in the single EICs, most molecules were detected as single deprotonated molecules ([M − H]^−^); that is, no analyte fragmentation was obvious, which is one of the advantages of the APCI ion source. Compared to that, an ionization with other commonly used techniques for GC-MS hyphenation, such as EI, normally leads to an intense fragmentation of sample molecules [39]. Thus, the developed GC-APCI-FT-ICR-MS hyphenation makes it easier to assign a molecular formula to the detected molecular ion. In contrast, GC-EI-MS simplifies the clarification of the exact structure of a compound, due to the higher number of detectable fragments with lower (*m/z*)-ratios. Hence, the combination of results from the developed GC-APCI-FT-ICR-MS hyphenation with results from GC-EI-MS hyphenation should increase the knowledge about pyrolysis oil to a greater extent. Moreover, an improved GC separation, for example, with an ionic liquid GC column or a high-temperature GC column, will also help to separate nonpolar and polar compounds similarly by using a higher GC maximum temperature. Thus, also molecules of higher molecular weight could be separated and analyzed. Furthermore, experiments involving collision-induced dissociation (CID) would allow the identification of specific compound structures or typical building units [40–42], especially of those contained in larger molecules.

## Supplementary Material

An overview of the detailed composition of the RMSC is given in the Supplementary Material, as well as a calibration list for the experiments, detailed figures of the data processing method, an overview of assigned compounds for alkyl phenols, alkyl dihydroxy benzenes and alkoxyl alkyl phenols with average (*m/z*) errors and *n_C_-DBE-t*-plots of all analyzed compound classes.

## Figures and Tables

**Table 1:** Assigned compounds for alkyl carboxylic acids, alkylene carboxylic acids, and dicarboxylic acids. Presumably, each identified compound is present as [M − H]^−^ molecular ion. Hence, calculated and observed (*m*/*z*)-ratios are presented in Da.

Assigned compound class	Assigned compound	Number	Retention time	(*m*/*z*)		(*m*/*z*)	
			*t*_*R*_	Calculated	Observed	Error	
			[min]	[Da]	[Da]	[mDa]	[ppm]
Alkyl carboxylic acids	Butanoic acid	1	12.55 ± 0.10	87.045153	87.045173	0.020	0.230
	Pentanoic acid	2	14.85 ± 0.06	101.060803	101.060824	0.021	0.208
	Hexanoic acid	3	17.19 ± 0.09	115.076453	115.076442	0.011	0.096
	Heptanoic acid	4	19.48 ± 0.12	129.092103	129.092084	0.019	0.147
	Octanoic acid	5	21.78 ± 0.09	143.107753	143.107720	0.033	0.231
	Nonanoic acid	6	24.16 ± 0.11	157.123403	157.123377	0.026	0.165
	Decanoic acid	7	26.46 ± 0.16	171.139053	171.139020	0.033	0.193
	Undecanoic acid	8	28.98 ± 0.15	185.154703	185.154677	0.026	0.140
	Dodecanoic acid	9	31.79 ± 0.23	199.170354	199.170269	0.085	0.427
	Tridecanoic acid	10	35.03 ± 0.09	213.186004	213.185954	0.050	0.235
	Tetradecanoic acid	11	38.63 ± 0.08	227.201654	227.201606	0.048	0.211
	Pentadecanoic acid	12	43.75 ± 0.12	241.217304	241.217230	0.074	0.307
	Hexadecanoic acid	13	50.93 ± 0.15	255.232954	255.232899	0.055	0.215
	Heptadecanoic acid	14	58.38 ± 0.02	269.248604	269.248546	0.058	0.215
	Octadecanoic acid	15	60.72 ± 0.03	283.264254	283.264212	0.042	0.148

Alkylene carboxylic acids	Hexenoic acid	16	18.56 ± 0.05	113.060803	113.060810	0.007	0.062
	Heptenoic acid	17	20.86 ± 0.09	127.076453	127.076468	0.015	0.118
	Octenoic acid	18	23.11 ± 0.17	141.092103	141.092100	0.003	0.021
	Nonenoic acid	19	25.26 ± 0.05	155.107753	155.107734	0.019	0.122
	Decenoic acid	20	27.75 ± 0.06	169.123403	169.123383	0.020	0.118
	Undecenoic acid	21	30.19 ± 0.07	183.139053	183.139030	0.023	0.126
	Dodecenoic acid	22	33.04 ± 0.15	197.154703	197.154674	0.029	0.147
	Tridecenoic acid	23	35.82 ± 0.16	211.170354	211.170287	0.067	0.317
	Tetradecenoic acid	24	39.69 ± 0.16	225.186004	225.185987	0.017	0.075
	Pentadecenoic acid	25	45.24 ± 0.06	239.201654	239.201572	0.082	0.343
	Hexadecenoic acid	26	51.32 ± 0.14	253.217304	253.217211	0.093	0.367

Dicarboxylic acids	Pentanedioic acid	27	31.94 ± 0.06	131.034982	131.034977	0.005	0.038
	Hexanedioic acid	28	33.04 ± 0.15	145.050632	145.050626	0.006	0.041
	Heptanedioic acid	29	35.72 ± 0.11	159.066282	159.066265	0.017	0.107
	Octanedioic acid	30	37.46 ± 0.18	173.081932	173.081916	0.016	0.092
	Nonanedioic acid	31	40.03 ± 0.05	187.097583	187.097570	0.013	0.069
	Decanedioic acid	32	43.87 ± 0.20	201.113233	201.113219	0.014	0.070
