# Supplementary material for: Gas Chromatography/Atmospheric Pressure Chemical Ionization-Fourier Transform Ion Cyclotron Resonance Mass Spectrometry of Pyrolysis Oil from German Brown Coal
Source: Int J Anal Chem. 2016 Mar 15;2016:5960916. doi: 10.1155/2016/5960916 (PMC4811094; doi:10.1155/2016/5960916)
Supplement: Supplementary file 1 — An overview of the detailed composition of the RMSC is given in the Supplementary Material, as well as a calibration list for the experiments, detailed figures of the data processing method, an overview of assigned compounds for alkyl phenols, alkyl dihydroxy benzenes and alkoxyl alkyl phenols with average (m/z) errors and nC-DBE-t-plots of all analyzed compound classes. [file 5960916.f1.pdf]

SUPPLEMENTARY MATERIAL

Gas Chromatography/Atmospheric Pressure Chemical Ionization-Fourier Transform Ion Cyclotron Resonance Mass Spectrometry of a Pyrolysis Oil from German Brown Coal

Jan Zuber[†], Marius M. Kroll[†], Philipp Rathsack[†,¶], Matthias Otto[†]

[†]*Institute of Analytical Chemistry, TU Bergakademie Freiberg, 09599 Freiberg, Germany*
[¶]*German Centre for Energy Resources, Reiche Zeche, Fuchsmuehlenweg 9, 09599 Freiberg, Germany*

Abstract

A pyrolysis oil from the slow pyrolysis of a German brown coal from Schöningen, obtained at a temperature of 500 °C, was separated and analyzed using a hyphenation of gas chromatography with an atmospheric pressure chemical ionization source operated in negative ion mode and Fourier transform ion cyclotron resonance mass spectrometry (GC-APCI-FT-ICR-MS). Development of this ultra-high-resolving analysis method is described, i.e. optimization of specific GC and APCI parameters and performed data processing. The advantages of GC-APCI-FT-ICR-MS hyphenation, e.g. soft ionization, ultra-high-resolving detection and most important isomer separation, were demonstrated for the sample liquid. For instance, it was possible to separate and identify nine different propylphenol, ethyldimethylphenol and trimethylphenol isomers. Furthermore, homologous series of different acids, e.g. alkyl and alkylene carboxylic acids, were verfied, as well as homologous series of alkyl phenols, alkyl dihydroxy benzenes and alkoxy alkyl phenols.

Keywords: coal, pyrolysis oil, FT-ICR-MS, APCI, GC-MS

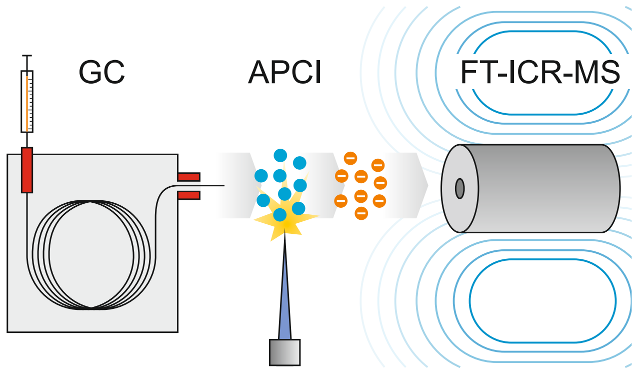

Contents

S1. Composition of the RMSC 3

S2. Calibration of GC-APCI-FT-ICR-MS analyses 4

S3. Data processing of GC-APCI-FT-ICR-MS analyses 5

S4. Compound assignment of alkyl phenols, alkyl dihydroxy phenols and alkoxy alkyl phenols 8

S5. Time-separated n_C-DBE-plots 12

List of Figures

Figure S1. Interface of the developed data processing and calibration tool. 5
Figure S2. Data structure of received peak lists. 6
Figure S3. Data structure of obtained molecular formula lists. 7
Figure S4. n_C-DBE-t-plots of different time segments and compound classes. 13

List of Tables

Table S1. Reference compounds of the RMSC. 3
Table S2. Calibration list for GC-APCI-FT-ICR-MS analyses. 4
Table S3. Assigned compounds for alkyl phenols. 9
Table S4. Assigned compounds for alkyl dihydroxy benzenes and alkoxy alkyl phenols. 10
Table S5. Calculated (m/z) errors (mean values) for assigned compound groups of alkyl phenols. . 10
Table S6. Calculated (m/z) errors (mean values) for assigned compound groups of alkyl dihydroxy benzenes and alkoxy alkyl phenols. 11

S1. Composition of the RMSC

Table S1. Reference compounds of the RMSC.

Reference compound	Molecular formula	Monoisotopic neutral mass [Da]
Benzene	C_6H_6	78.046950
2-Cyclopenten-1-one	C_5H_6O	82.041865
(5H)-Furan-2-one	$C_4H_4O_2$	84.021129
Cyclopentanone	C_5H_8O	84.057515
Toluene	C_7H_8	92.062600
Phenol	C_6H_6O	94.041865
Furfural	$C_5H_4O_2$	96.021129
2-Methyl-2-cyclopenten-1-one<br>3-Methyl-2-cyclopenten-1-one	C_6H_8O	96.057515
2-Methylthiophene<br>3-Methylthiophene	C_5H_6S	98.019021
2-Furfuryl alcohol	$C_5H_6O_2$	98.036779
Styrene	C_8H_8	104.062600
1,2-Dimethylbenzene<br>1,4-Dimethylbenzene<br>Ethylbenzene	C_8H_{10}	106.078250
o-Cresol<br>*m*-Cresol	C_7H_8O	108.057515
2-Acetylfurane<br>Catechol<br>Hydroquinone	$C_6H_6O_2$	110.036779
2,3-Dimethyl-2-cyclopenten-1-one	$C_7H_{10}O$	110.073165
2-Ethylthiophene	C_6H_8S	112.034671
Indene	C_9H_8	116.062600
Benzofurane	C_8H_6O	118.041865
2,5-Dimethylphenol<br>2,6-Dimethylphenol<br>3,5-Dimethylphenol<br>2-Ethylphenol<br>3-Ethylphenol<br>4-Ethylphenol	$C_8H_{10}O$	122.073165
3-Methylcatechol<br>4-Methylcatechol<br>2-Methylhydroquinone<br>Guaiacol<br>Mequinol	$C_7H_8O_2$	124.052429
Naphthalene	$C_{10}H_8$	128.062600
Benzo*[b]*thiophene	C_8H_6S	134.019021
4-Methylguaiacol	$C_8H_{10}O_2$	138.068080
1-Methylnaphthalene<br>2-Methylnaphthalene	$C_{11}H_{10}$	142.078250
2-Methylbenzo*[b]*thiophene<br>3-Methylbenzo*[b]*thiophene	C_9H_8S	148.034671
Vanillin	$C_8H_8O_3$	152.047344
4-Ethylguaiacol	$C_9H_{12}O_2$	152.083730
Syringol	$C_8H_{10}O_3$	154.062994
Isoeugenol (*cis*)<br>Isoeugenol (*trans*)<br>Eugenol	$C_{10}H_{12}O_2$	164.083730
4-Propylguaiacol	$C_{10}H_{14}O_2$	166.099380

S2. Calibration of GC-APCI-FT-ICR-MS analyses

Table S2 shows the molecules and their individual (m/z)-ratios, used for calibration of GC-APCI-FT-ICR-MS single mass spectra.

Table S2. Calibration list for GC-APCI-FT-ICR-MS analyses. Presumably, each compound can be detected as $[M - H]^-$ molecular ion.

Molecular formula	(m/z) calculated	Assigned compound
$C_2F_3O_2$	112.985587	Trifluoroacetic acid (TFA)
$C_6H_{11}O_3$	131.071368	Polyethylene glycol (PEG) trimer
C_3F_7	168.989371	Decarboxylated heptafluorobutyric acid (HFBA)
$C_4F_7O_2$	212.979200	HFBA
$C_{15}H_{23}O$	219.175439	Butylated hydroxytoluene (BHT)
$C_{15}H_{21}O_2$	233.154703	3,5-Di-*tert*-butylsalicylaldehyde
$C_{16}H_{31}O_2$	255.232954	Palmitic (Hexadecanoic) acid
$C_{18}H_{35}O_2$	283.264254	Stearic (Octadecanoic) acid

According to Table S2, molecular ions for data calibration were used, that descend from calibration compounds and from actual sample compounds. For instance $C_2F_3O_2$, C_3F_7 and $C_4F_7O_2$ are ions, that can be affiliated to the liquid calibration standard solution. Furthermore, $C_6H_{11}O_3$ refers to a PEG trimer, which elutes out of the GC along with the sample molecules. The installed SUPELCOWAX 10 capillary column has PEG bond as stationary phase. Therefore, the stationary phase is probably the source for this calibrant molecular ion.

Other ions, used for calibration, can be affiliated to the actual liquid oil sample. For example $C_{15}H_{23}O$ refers to butylated hydroxytoluene (BHT), a compound normally used as THF stabilizer [43]. Hence, its discharge arises from the pyrolysis process, where gaseous and liquid pyrolysis products were trapped in cooled traps. One trap was filled with tetrahydrofuran (THF), thus, a discharge into the sample liquids was unavoidable. 3,5-Di-*tert*-butylsalicylaldehyde ($C_{15}H_{21}O_2$) is most likely a product of BHT, that is formed over time out of the THF stabilizer. Formulas $C_{16}H_{31}O_2$ and $C_{18}H_{35}O_2$ can be assigned to palmitic (hexadecanoic) and stearic (octadecanoic) acid, compounds typically found in the pyrolysis oils, as shown in the main part of the paper.

S3. Data processing of GC-APCI-FT-ICR-MS analyses

In the following figures, three different screen shots of the developed calibration and data processing tool and its resulting files are illustrated. Figure S1 displays the interface of the tool, Figure S2 the data structure of received peak lists and Figure S3 the data structure of obtained molecular formula lists.

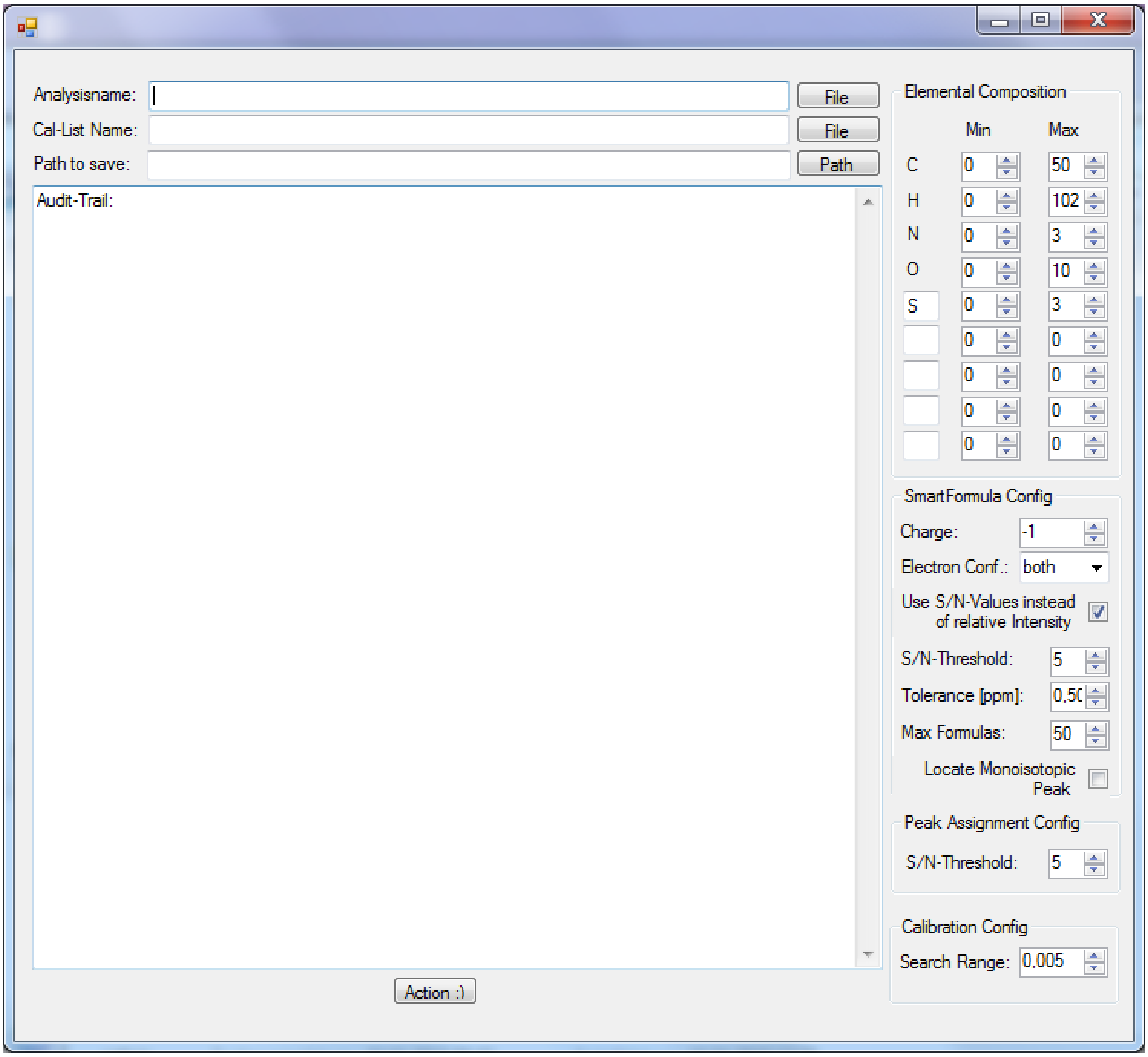

Figure S1. Interface of the developed data processing and calibration tool. Despite of some general settings, like adjustment of assumed numbers for C, H, N, O, S atoms and other elements (*Elemental Composition*) for the molecular formula prediction, it was possible to change more specific settings, e.g. presumed molecular ion charge (*Charge*), electron configuration (*Electron. Conf.*), *S/N* threshold (*S/N-Threshold*), (m/z) tolerance in ppm (*Tolerance [ppm]*) and maximum number of formulas (*Max. Formulas*). Furthermore, the search range (in ppm) for molecular ions used for data calibration (*Calibration Config. - Search Range*) can be adjusted. Resulting output files are presented in the following two figures.

00100_peak.csv

```
m/z, I, Res., S/N
95.787749, 586926, 306632, 31.1
106.489713, 589529, 243461, 31.2
112.985591, 965392, 265416, 51.1
130.041086, 116673, 225368, 6.2
133.040821, 106699, 251576, 5.6
141.045889, 118181, 220718, 6.3
142.041136, 165553, 232601, 8.8
144.045543, 101098, 238242, 5.4
144.056764, 111435, 220398, 5.9
145.040786, 113970, 198014, 6.0
149.108458, 102213, 194865, 5.4
155.036413, 141913, 206238, 7.5
155.061654, 105941, 179716, 5.6
156.056762, 170859, 180285, 9.0
157.052035, 97692, 222384, 5.2
158.036068, 100613, 207202, 5.3
159.056443, 106380, 207814, 5.6
160.051700, 120019, 200494, 6.4
162.982496, 234161, 175861, 12.4
165.046046, 99931, 155379, 5.3
166.041162, 150826, 177311, 8.0
167.036324, 112443, 191953, 6.0
168.056744, 167940, 199571, 8.9
168.989401, 2655317, 172888, 140.5
169.040903, 124876, 196375, 6.6
169.052190, 121542, 136570, 6.4
170.072445, 142317, 168664, 7.5
171.056438, 144864, 179697, 7.7
171.067778, 110185, 168675, 5.8
172.051757, 158493, 175682, 8.4
172.088173, 101368, 150386, 5.4
173.108446, 101178, 192897, 5.4
174.067408, 115122, 176481, 6.1
174.128960, 203959, 158581, 10.8
```

Figure S2. Data structure of received peak lists. For one time segment (here: *00100*) all detected compounds with their individual (m/z)-ratio ((m/z)), intensity (I), resolution (*Res.*) and their S/N are listed.

00100_sf.csv

```
mSigma;Observed Intens;Observed m/z;calc. m/z;err mDa;err ppm;sum formula;C;H;N;O;S;
0.781735599040985;116673;130.041086;130.041071;-0.115006431937218;-0.88438535665455;C 7 H 4 N 3 ;7;4;3;0;0;
-1;111435;144.056764;144.056721;-0.296615749597549;-2.05901994712051;C 8 H 6 N 3 ;8;6;3;0;0;
0.772187173366547;102213;149.108458;149.108422;-0.242828786373138;-1.62853797350803;C 9 H 13 N 2 ;9;13;2;0;0;
-1;170859;156.056762;156.056721;0.262199014425278;1.68015157793352;C 9 H 6 N 3 ;9;6;3;0;0;
-1;167940;168.056744;168.056721;0.139951229095459;0.832761753614808;C 10 H 6 N 3 ;10;6;3;0;0;
-1;101178;173.108446;173.108422;0.141242563724518;0.815919538390899;C 11 H 13 N 2 ;11;13;2;0;0;
-1;111052;177.066974;177.066951;0.129663705825806;0.7322862239112;C 9 H 9 N 2 O 2 ;9;9;2;2;0;
0.760075867176056;449221;177.128537;177.128489;-0.270052969455719;-1.52461582233904;C 12 H 17 O 1 ;12;17;0;1;0;
-1;108454;184.088038;184.088021;-0.0926244929432869;-0.50315324106153;C 11 H 10 N 3 ;11;10;3;0;0;
-1;178265;186.067299;186.067285;-0.0729145556688309;-0.391871951955175;C 10 H 8 N 3 O 1 ;10;8;3;1;0;
-1;95539;189.066927;189.066951;-0.125679165124893;-0.66473373679252;C 10 H 9 N 2 O 2 ;10;9;2;2;0;
0.761792778968811;103864;189.103381;189.103337;-0.234501749277115;-1.240071690273;C 11 H 13 N 2 O 1 ;11;13;2;1;0;
-1;147447;196.088078;196.088021;-0.290449261665344;-1.48121836280074;C 12 H 10 N 3 ;12;10;3;0;0;
-1;104819;198.056028;198.056052;-0.12234391272068;-0.617723752461014;C 12 H 8 N 1 O 2 ;12;8;1;2;0;
0.404416531324387;160286;198.067294;198.067285;0.0448347181081772;0.22636103681402;C 11 H 8 N 3 O 1 ;11;8;3;1;0;
-1;179095;199.124074;199.124072;-0.00821307860314846;-0.0412460354506181;C 13 H 15 N 2 ;13;15;2;0;0;
-1;99124;200.082927;200.082936;0.0442197732627392;0.221007229327096;C 11 H 10 N 3 O 1 ;11;10;3;1;0;
-1;145967;201.139758;201.139722;0.179621890187263;0.893020314520222;C 13 H 17 N 2 ;13;17;2;0;0;
-1;186317;206.072411;206.072371;-0.193684577941895;-0.939886019792259;C 13 H 8 N 3 ;13;8;3;0;0;
-1;126054;208.051664;208.051635;0.136304780840874;0.655148718229007;C 12 H 6 N 3 O 1 ;12;6;3;1;0;
-1;142983;209.118314;209.118318;-0.0210807491093874;-0.100807761639611;C 12 H 17 O 3 ;12;17;0;3;0;
-1;121074;210.067271;210.067285;-0.0694272965192795;-0.330500302243406;C 12 H 8 N 3 O 1 ;12;8;3;1;0;
-1;124715;211.087628;211.087687;-0.278684496879578;-1.32023131753345;C 13 H 11 N 2 O 1 ;13;11;2;1;0;
-1;106032;212.082982;212.082936;-0.218894734978676;-1.03211833857369;C 12 H 10 N 3 O 1 ;12;10;3;1;0;
-1;108250;212.119291;212.119321;0.142400398850441;0.671322246497539;C 13 H 14 N 3 ;13;14;3;0;0;
0.210417628288269;214202;212.983768;212.983816;-0.224771603941917;-1.05534617117718;C 12 H 5 S 2 ;12;5;0;0;2;
-1;125032;213.066956;213.066951;0.0251579098403454;0.118075135892752;C 12 H 9 N 2 O 2 ;12;9;2;2;0;
-1;105471;214.062258;214.0622;0.269672065973282;1.25978333931396;C 11 H 8 N 3 O 2 ;11;8;3;2;0;
-1;135985;214.135031;214.134971;-0.277444183826447;-1.29565061426599;C 13 H 16 N 3 ;13;16;3;0;0;
-1;105780;214.983462;214.983519;0.262972593307495;1.22322243190579;C 14 H 1 N 1 S 1 ;14;1;1;0;1;
-1;136613;215.118924;215.118987;-0.292231559753418;-1.35846514363541;C 13 H 15 N 2 O 1 ;13;15;2;1;0;
0.398084223270416;139575;216.114266;216.114236;0.142101913690567;0.657531388720266;C 12 H 14 N 3 O 1 ;12;14;3;1;0;
-1;170295;217.134622;217.134637;-0.0658299177885056;-0.303175592370303;C 13 H 17 N 2 O 1 ;13;17;2;1;0;
-1;135582;218.072434;218.072371;0.291711330413818;1.33768090006141;C 14 H 8 N 3 ;14;8;3;0;0;
```

Figure S3. Data structure of obtained molecular formula lists. Contained in this file is an information about the standard deviation (*mSigma*), observed signal intensity (*Observed Intens.*), observed (*m/z*)-ratio of the detected molecular ion (*Observed m/z*), theoretical calculated (*m/z*)-ratio (*calc. m/z*), mass error in mDa (*err mDa*), mass error in ppm (*err ppm*), the assigned molecular formula (*sum formula*) and the elements comprised in this molecular formula (here: *C, H, N, O, S*) in the time segment (here: *00100*).

S4. Compound assignment of alkyl phenols, alkyl dihydroxy phenols and alkoxy alkyl phenols

In the following tables, an overview for allocated alkyl phenols (see Table S3) and alkyl dihydroxy benzenes/alkoxy alkyl phenols (see Table S4) is presented. Assigned compounds, according to the RMSC, are specified in these tables as well. Calculated (m/z) errors varied in case of the alkyl phenols from 0.007 to 0.439 ppm (mean value: 0.088 ppm) and in case of the alkyl dihydroxy benzenes/alkoxy alkyl phenols from 0.000 to 0.471 ppm (mean value: 0.124 ppm). Hence, the applied assignment of observed (m/z)-ratios to the described oil compounds is most accurate for the alkyl phenols, but also relatively accurate for the alkyl dihydroxy benzenes/alkoxy alkyl phenols.

Furthermore, described (m/z) error ranges and mean values for each individual compound group of alkyl phenols and alkyl dihydroxy benzenes/alkoxy alkyl phenols are listed in Table S5 and Table S6. According to the results presented, compound assignment of heptyl phenols (and isomers) is most precise in the compound class of alkyl phenols. Compared to that, compound assignment of dihydroxy tetramethyl benzenes (and isomers) is most accurate in compound class alkyl dihydroxy benzenes/alkoxy alkyl phenols. Notable in compound class alkyl dihydroxy benzenes/alkoxy alkyl phenols, is the exact assignment of two compounds of dihydroxy dimethyl benzenes (and isomers), comparing observed and calculated (m/z) errors.

Table S3. Assigned compounds for alkyl phenols. Presumably, each supposed compound is present as $[M-H]^-$ molecular ion. Hence, calculated and observed (m/z)-ratios are presented in Da.

**Assigned compound class**	**Assigned compound**	**No.**	**Retention time t_R [min]**	**(m/z) calculated [Da]**	**observed [Da]**	**(m/z) error [mDa]**	**[ppm]**
Phenols	Phenol	a	20.38 ± 0.08	93.034588	93.034599	0.011	0.118
Methyl phenols	*o*-Cresol	b	20.25 ± 0.05	107.050238	107.050246	0.008	0.075
	m-Cresol	c	22.09 ± 0.05	107.050238	107.050240	0.002	0.019
Dimethyl phenols (and isomers)	2,6-Dimethylphenol	d	18.47 ± 0.05	121.065888	121.065895	0.007	0.058
	2-Ethylphenol	e	21.95 ± 0.07	121.065888	121.065876	0.012	0.099
			23.34 ± 0.04	121.065888	121.065892	0.004	0.033
			23.94 ± 0.05	121.065888	121.065853	0.035	0.289
			24.80 ± 0.08	121.065888	121.065899	0.011	0.091
Trimethyl phenols (and isomers)			19.41 ± 0.05	135.081539	135.081549	0.010	0.074
			21.29 ± 0.07	135.081539	135.081552	0.013	0.096
			22.65 ± 0.06	135.081539	135.081553	0.014	0.104
			23.23 ± 0.06	135.081539	135.081543	0.004	0.030
			23.73 ± 0.05	135.081539	135.081544	0.005	0.037
			24.94 ± 0.05	135.081539	135.081544	0.005	0.037
			25.72 ± 0.07	135.081539	135.081521	0.018	0.133
			26.41 ± 0.05	135.081539	135.081545	0.006	0.044
			27.77 ± 0.07	135.081539	135.081535	0.004	0.030
Tetramethyl phenols (and isomers)			20.09 ± 0.04	149.097189	149.097190	0.001	0.007
			20.70 ± 0.05	149.097189	149.097163	0.026	0.174
			21.24 ± 0.12	149.097189	149.097188	0.001	0.007
			22.27 ± 0.07	149.097189	149.097200	0.011	0.074
			22.78 ± 0.05	149.097189	149.097174	0.015	0.101
			23.49 ± 0.07	149.097189	149.097173	0.016	0.107
			24.10 ± 0.08	149.097189	149.097186	0.003	0.020
			24.76 ± 0.09	149.097189	149.097195	0.006	0.040
			25.38 ± 0.13	149.097189	149.097199	0.010	0.067
			26.57 ± 0.04	149.097189	149.097174	0.015	0.101
			27.25 ± 0.03	149.097189	149.097181	0.008	0.054
			28.09 ± 0.11	149.097189	149.097193	0.004	0.027
			29.23 ± 0.03	149.097189	149.097197	0.008	0.054
Pentamethyl phenols (and isomers)			22.81 ± 0.11	163.112839	163.112864	0.025	0.153
			23.63 ± 0.08	163.112839	163.112816	0.023	0.141
			24.56 ± 0.11	163.112839	163.112819	0.020	0.123
			25.27 ± 0.09	163.112839	163.112813	0.026	0.159
			25.95 ± 0.11	163.112839	163.112828	0.011	0.067
			27.77 ± 0.07	163.112839	163.112819	0.020	0.123
			28.84 ± 0.05	163.112839	163.112858	0.019	0.116
			30.50 ± 0.15	163.112839	163.112823	0.016	0.098
Hexyl phenols (and isomers)			26.79 ± 0.04	177.128489	177.128479	0.010	0.056
			27.77 ± 0.07	177.128489	177.128505	0.016	0.090
			29.56 ± 0.07	177.128489	177.128492	0.003	0.017
			29.87 ± 0.04	177.128489	177.128497	0.008	0.045
			30.57 ± 0.13	177.128489	177.128499	0.010	0.056
Heptyl phenols (and isomers)			30.01 ± 0.13	191.144139	191.144133	0.006	0.031
			30.56 ± 0.10	191.144139	191.144147	0.008	0.042
			32.12 ± 0.07	191.144139	191.144146	0.007	0.037
			32.44 ± 0.04	191.144139	191.144131	0.008	0.042
Octyl phenols (and isomers)			27.01 ± 0.09	205.159789	205.159740	0.049	0.239
			33.72 ± 0.16	205.159789	205.159759	0.030	0.146
			33.96 ± 0.11	205.159789	205.159699	0.090	0.439

Table S4. Assigned compounds for alkyl dihydroxy benzenes and alkoxy alkyl phenols. Most likely, each supposed compound is present as $[M - H]^-$ molecular ion as well.

**Assigned compound class**	**Assigned compound**	**No.**	**Retention time t_R [min]**	**(m/z) calculated [Da]**	**(m/z) observed [Da]**	**(m/z) error [mDa]**	**(m/z) error [ppm]**
Dihydroxy benzenes			20.35 ± 0.08	109.029503	109.029519	0.016	0.147
	Hydroquinone	f	34.60 ± 0.12	109.029503	109.029458	0.045	0.413
Dihydroxy methyl benzenes (and isomers)	Guaiacol	g	17.44 ± 0.02	123.045153	123.045167	0.014	0.114
			22.11 ± 0.05	123.045153	123.045154	0.001	0.008
	4-Methylcatechol	h	33.86 ± 0.11	123.045153	123.045095	0.058	0.471
	2-Methylhydroquinone	i	37.19 ± 0.12	123.045153	123.045101	0.052	0.423
Dihydroxy dimethyl benzenes (and isomers)	4-Methylguaiacol	j	19.53 ± 0.10	137.060803	137.060803	0.000	0.000
			21.94 ± 0.08	137.060803	137.060796	0.007	0.051
			23.88 ± 0.09	137.060803	137.060803	0.000	0.000
			32.69 ± 0.10	137.060803	137.060795	0.008	0.058
			36.28 ± 0.12	137.060803	137.060776	0.027	0.197
			41.22 ± 0.05	137.060803	137.060767	0.036	0.263
Dihydroxy trimethyl benzenes (and isomers)	4-Ethylguaiacol	k	21.05 ± 0.08	151.076453	151.076451	0.002	0.013
			23.22 ± 0.09	151.076453	151.076438	0.015	0.099
			23.77 ± 0.08	151.076453	151.076432	0.021	0.139
			24.82 ± 0.08	151.076453	151.076434	0.019	0.126
			25.76 ± 0.18	151.076453	151.076471	0.018	0.119
			33.94 ± 0.16	151.076453	151.076458	0.005	0.033
			40.05 ± 0.05	151.076453	151.076460	0.007	0.046
			46.45 ± 0.13	151.076453	151.076442	0.011	0.073
Dihydroxy tetramethyl benzenes (and isomers)	4-Propylguaiacol	l	22.70 ± 0.13	165.092103	165.092093	0.010	0.061
			34.30 ± 0.17	165.092103	165.092092	0.011	0.067
			36.32 ± 0.12	165.092103	165.092102	0.001	0.006
			42.46 ± 0.08	165.092103	165.092094	0.009	0.055

Table S5. Calculated (m/z) errors (mean values) for assigned compound groups of alkyl phenols.

**Assigned compound class**	**(m/z) error (mean value) [mDa]**	**(m/z) error (mean value) [ppm]**
Phenol	0.011	0.118
Methyl phenols	0.005	0.047
Dimethyl phenols (and isomers)	0.014	0.114
Trimethyl phenols (and isomers)	0.009	0.065
Tetramethyl phenols (and isomers)	0.010	0.064
Pentamethyl phenols (and isomers)	0.020	0.123
Hexyl phenols (and isomers)	0.009	0.053
Heptyl phenols (and isomers)	0.007	0.038
Octyl phenols (and isomers)	0.056	0.275
**Average**	**0.014**	**0.088**

Table S6. Calculated (m/z) errors (mean values) for assigned compound groups of alkyl dihydroxy benzenes and alkoxy alkyl phenols.

**Assigned compound class**	**(m/z) error (mean value) [mDa]**	**(m/z) error (mean value) [ppm]**
Dihydroxy benzenes	0.030	0.280
Dihydroxy methyl benzenes (and isomers)	0.031	0.254
Dihydroxy dimethyl benzenes (and isomers)	0.013	0.095
Dihydroxy trimethyl benzenes (and isomers)	0.012	0.081
Dihydroxy tetramethyl benzenes (and isomers)	0.008	0.047
**Average**	**0.016**	**0.124**

S5. Time-separated n_C-DBE-plots

In Figure S4, the n_C-DBE-t-plots of all evaluated compound classes of liquid sample S500 are presented, color-coded to the relative intensity (yellow - low intensity, red - medium intensity, black - high intensity). Interpretation of these plots for classes $C_cH_hO_1$, $C_cH_hO_2$, $C_cH_hO_4$ and $C_cH_hO_2S_1$ was conducted in the section "Results and Discussion" of the paper. Other classes, that were not evaluated in the main part of the paper, will be analyzed hereafter.

First compound class to evaluate is compound class $C_cH_hO_3$. In this compound class, increasing n_C and/or DBE values with advancing analysis time were not noticed. Compounds of lower DBE as well as compounds of higher DBE were detected throughout the analysis. In compound class $C_cH_hO_5$, a decrease of observed n_C as well as DBE values with advancing analysis time was obvious. Therefore, presumably oxygen-functionalized benzene derivatives with DBE values between 4 and 7 mainly eluted in a time range of 10 - 40 min.

The time-depending n_C-DBE-plot of sulphur-containing compound class $C_cH_hS_1$ expressed a time-dependent elution and thus detection of supposed cycloalkane substituted thiophenes ($DBE = 4$) and benzothiophenes ($DBE = 7$). More precisely, presumed thiophenic compounds were detected in a time range of 10 - 30 min and benzothiophenic compounds between 30 and 50 min. In comparison, presumed hydroxy functionalized thiophenes and benzothiophenes with bonded cycloalkanes ($DBE = 4$ and $DBE = 7$) were detected in class $C_cH_hO_1S_1$ in a time range between 10 and 40 min and 20 - 60 min. In general, an increase of n_C and DBE values was noticed in this compound class, especially between 10 and 60 min.

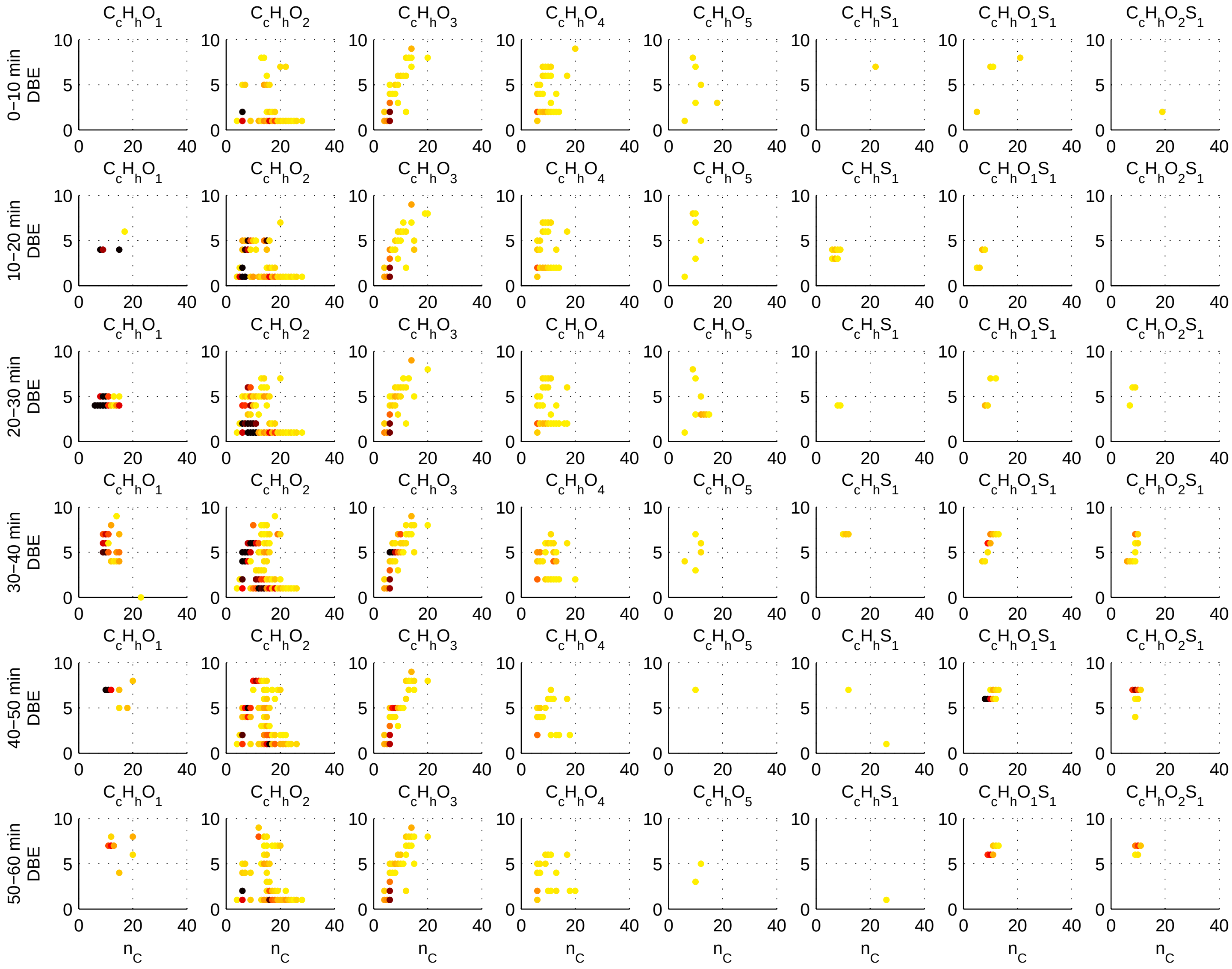

Figure S4. n_C-DBE-t-plots of different time segments and compound classes.
